# Posterior Scleritis with Inflammatory Retinal Detachment

**DOI:** 10.5811/westjem.2015.8.28349

**Published:** 2015-12-14

**Authors:** Jesse Z. Kellar, Brian T. Taylor

**Affiliations:** Lakeland Health, Department of Emergency Medicine, St. Joseph, Michigan

## Abstract

A 14-year-old African American male presented to the emergency department with worsening left eye redness, swelling, and vision loss over the preceding three days. History was notable for similar eye redness and swelling without vision loss four months earlier, which improved following a brief course of prednisone. He endorsed mild eye irritation and tearing with bright lights. There was no history of fever, respiratory symptoms or trauma. Mother was medicating patient with leftover antibiotic eye drops x3 days without improvement. Physical examination on presentation notable for proptosis of left eye, lid, and periorbital swelling, mild scleral injection, and central vision loss in affected eye (20/200 OS, 20/25 OD). Extraocular movements and pupillary exam were normal. No corneal fluorescein uptake, abnormal cell, flare, or siedel sign were seen during slit lamp exam. Eye pressures were 24 mmHg in both eyes. Bedside ultrasonography was performed (Figure 1 showing retinal detachment, Ultrasound Video 2 showing detachment in orbital scan).

## DIAGNOSIS

### Posterior Scleritis with Inflammatory Retinal Detachment

Scleritis is a potentially sight-threatening underdiagnosed inflammatory disease affecting the sclera of the eye.^1^ Of the five categories of scleritis described by the Watson System (diffuse anterior, nodular anterior, necrotizing anterior without inflammation, necrotizing anterior with inflammation, and posterior scleritis), posterior scleritis is the most rare, accounting for only 2% to 12% of all cases. Because the average age of patients with posterior scleritis is 45 to 49 years, posterior scleritis in children is even rarer.^2^–^4^ The ophthalmic literature consists of predominantly single case reports. Scleritis can be the first symptom of an onset of connective tissue systemic diseases, but in children often no systemic association is found.^1^

Decreased vision can be a presenting sign, although normal vision can still be present. Other symptoms include, but are not limited to, periocular pain, headache, and pain with extraocular movement.^3^ Signs of physical examination may include concurrent ciliary or conjunctival injection, anterior uveitis, disc swelling, serous thickening, detachment of the retina, retinal striae, proptosis, and limitation of extraocular motility.^1^

Diagnosis is typically arrived at using a combination of clinical features and demonstration of scleral thickening (T-sign) on B-scan ultrasonography (tool used by ophthalmologists). Our patient underwent an optical coherence tomography scan confirming serous detachment over the macula.

First-line treatment includes topical steroid and oral nonsteroidal anti-inflammatory drugs. Systemic corticosteroids are added if first-line therapy is ineffective, and are adjusted to clinical response. Secondary immunosuppressive agents are sometimes used if symptoms are not adequately controlled. Long-term suppression is often required to prevent recurrence, and visual outcome is favorable.^1^

## Figures and Tables

**Figure:**
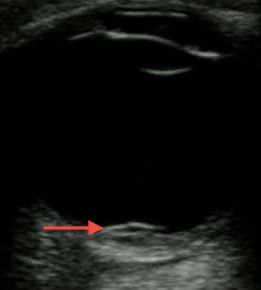
Ultrasound of left eye demonstrating a small flap irregularity on posterior retina near optic nerve indicating the retinal detachment (red arrow).

**Video:** Video of retinal detachment. Scan of left eye showing the retinal detachment, with audio commentary.
